# Identification of a lipid homeostasis-related gene signature for predicting prognosis, immunity, and chemotherapeutic effect in patients with gastric cancer

**DOI:** 10.1038/s41598-024-52647-7

**Published:** 2024-02-05

**Authors:** Chao Li, Zhen Xiong, Jinxin Han, Weiqi Nian, Zheng Wang, Kailin Cai, Jinbo Gao, Guobin Wang, Kaixiong Tao, Ming Cai

**Affiliations:** 1 Department of Gastrointestinal Surgery, Union Hospital, Tongji Medical College, Huazhong University of Science and Technology, Wuhan, China; 2 Department of Oncology, Chongqing Hospital of Traditional Chinese Medicine, Chongqing, China

**Keywords:** Cancer, Computational biology and bioinformatics, Immunology, Gastroenterology, Oncology

## Abstract

Gastric cancer (GC) is one of the most common and deadliest cancers worldwide. Lipid homeostasis is essential for tumour development because lipid metabolism is one of the most important metabolic reprogramming pathways within tumours. Elucidating the mechanism of lipid homeostasis in GC might significantly improve treatment strategies and patient prognosis. GSE62254 was applied to construct a lipid homeostasis-related gene signature score (HGSscore) by multiple bioinformatic algorithms including weighted gene coexpression network analysis (WGCNA) and LASSO-Cox regression. A nomogram based on HGSscore and relevant clinical characteristics was constructed to predict the survival of patients with GC. TIMER and xCell were used to evaluate immune and stromal cell infiltration in the tumour microenvironment. Correlations between lipid homeostasis-related genes and chemotherapeutic efficacy were analysed in GSCAlite. RT‒qPCR and cell viability assays were applied to verify the findings in this study. HGSscore was constructed based on eighteen lipid homeostasis-related genes that were selected by WGCNA and LASSO-Cox regression. HGSscore was strongly associated with advanced TNM stage and showed satisfactory value in predicting GC prognosis in three independent cohorts. Furthermore, we found that HGSscore was associated with the tumour mutation burden (TMB) and immune/stromal cell infiltration, which are related to GC prognosis, indicating that lipid homeostasis impacts the formation of the tumour microenvironment (TME). With respect to the GSCAlite platform, *PLOD2* and *TGFB2* were shown to be positively related to chemotherapeutic resistance, while *SLC10A7* was a favourable factor for chemotherapy efficacy. Cell viability assays showed that disrupted lipid homeostasis could attenuate GC cell viability. Moreover, RT‒qPCR revealed that lipid homeostasis could influence expression of specific genes. We identified a lipid homeostasis-related gene signature that correlated with survival, clinical characteristics, the TME, and chemotherapeutic efficacy in GC patients. This research provides a new perspective for improving prognosis and guiding individualized chemotherapy for patients with GC.

## Introduction

Gastric cancer (GC) is a significant health concern in eastern Asia, and GC generally has poor prognosis because it is often diagnosed at an advanced stage^1^. The TNM stage is still by far the most commonly used prognostic indicator. Nevertheless, several studies have demonstrated that the TNM staging system is insufficient for recent clinical treatment of GC patients^2,3^. Therefore, exploring a new prognostic tool for gastric cancer is essential.

Lipids are a complex superfamily that includes phospholipids, fatty acids, and cholesterol. These biomolecules not only constitute the basis of biological membranes but also participate in the energy supply process and intracellular signal transduction. In our previous study, we found that fatty acids derived from autophagy were needed for cancer cell survival^4^. Moreover, we revealed that the lipid metabolism patterns of GC tissues differ from those of normal stomach tissues, which is a risk factor for patient survival^5^. In addition, it has been reported that altered lipid metabolism is among the most prominent metabolic alterations in cancer^6^, and lipid homeostasis is crucial for maintaining cancer cell malignancy^7^. We are interested in determining whether lipid homeostasis can predict the prognosis of GC patients.

In this study, we demonstrated the importance of lipid homeostasis in GC and further explored a series of genes associated with the expression pattern of lipid homeostasis. Additionally, we defined this gene set as the lipid homeostasis-related gene signature score (HGSscore) and evaluated its prognostic value in three independent GC cohorts. Finally, we analysed correlations between HGSscore and immune/stromal cell infiltration and between HGSscore and chemotherapeutic efficacy.

## Materials and methods

### Cohorts and data processing

For confidence in this study, three independent cohorts involving sufficient cases were analysed in this research, namely, GSE62254^8^ and GSE84437^9^ from the Gene Expression Omnibus (GEO) database and TCGA-STAD from The Cancer Genome Atlas (TCGA). The GSE62254 dataset was used as the training cohort to construct the prognostic prediction model, and external validation was performed in the other two cohorts. All the data were analysed with R software.

### Single sample gene set enrichment analysis

The package “GSVA” in R software was used to perform single-sample gene set enrichment analysis (ssGSEA). ssGSEA was used to explore the expression patterns of genes related to lipid homeostasis and other tumour-related pathways (Supplementary Table S1) for analysis. Tumour-related pathway information was obtained from MsigDB.

### Weighted gene coexpression network analysis

Weighted gene coexpression network analysis (WGCNA) was performed based on the whole-transcriptome profile of GSE62254. After eliminating the genes above the cut-off height, we selected 3 as the best soft threshold, and the minimum number of genes within a module was 60. The module that was significantly associated with lipid homeostasis was retained for further study.

### Establishment and validation of HGSscore based on the LASSO-Cox algorithm

Using univariate Cox regression, overall survival (OS)-related genes were identified from the selected module. Subsequently, LASSO-Cox regression was conducted to determine genes highly correlating with patient prognosis and their correlation coefficients. The above parameters were used to calculate the lipid homeostasis-related gene signature score (HGSscore), which was calculated as Σ(coefficient * gene expression). Next, the HGSscore was validated for various clinical characteristics, such as TNM stage, OS, and disease-free survival (DFS), in all patients. The R packages “survival” and “rms” were applied to construct a nomogram based on HGSscore and other clinical characteristics. Finally, 5-, 6-, and 7-year survival probabilities were estimated by calibration curves.

### External validation of HGSscore

The Xtile program was used to distinguish HGSscore-high and -low groups in the validation cohort. K‒M OS curves were generated to determine the prognostic differences between HGSscore-high and -low groups in the GSE84437 and TCGA-STAD cohorts. In addition, we conducted multivariate Cox regression to validate the efficiency of the model.

### Infiltration of immune and stromal cells

We used the TIMER online server to estimate correlations between tumour-infiltrating immune cells and the highest 2 risk and protective genes, *TGFB2*, *UBE2D1*, *NAB2*, and *ASPRV1*. The xCell^10^ algorithm was subsequently used to evaluate infiltration of various stromal and immune cells in the TME. The ESTIMATE algorithm was applied to assess the tumour purity of the GC tissue.

### Prediction of chemotherapeutic effect

The GSCAlite^11^ platform was used to predict chemotherapeutic efficacy. The relationship between HGSscore and chemotherapeutic efficacy was evaluated by Spearman correlation analysis.

### Cell viability and RT‒qPCR

SGC7901 cells were incubated in DMEM containing 10% FBS (Promega). For the cell viability assay, 10,000 cells were seeded in 96-well plates overnight. Then, the cells were exposed to various lipids (Aladdin), including unsaturated fatty acids such as arachidonic acid (AA), α-linolenic acid (ALA), docosahexaenoic acid (DHA), eicosapentaenoic acid (EPA), linolenic acid (LA), and γ-linolenic acid (γLA), and saturated fatty acids such as C15, 17, and C18 at 20 µM for 24 h. Cell viability was evaluated using a CCK8 assay (Biosharp). For the RT‒qPCR assay, 600,000 cells were seeded in 6-well plates. The next day, the cells were treated with ethanol and 20 µM γLA for 24 h. Total mRNA was extracted with RNAiso Plus (Takara) and reverse transcribed to cDNA. For each sample, a total volume of 20 µl was used, containing 4 µl of mRNA, 6 µl of Master Mix (Takara) and 10 µl of RNase-free H_2_O, and the mixture was diluted to 160 µl after the reaction. Finally, we performed RT‒qPCR using TB Green (Takara) and a StepOne Real-Time PCR System in a reaction including 4 µl of cDNA, 5 µl of TB Green II, 0.8 µl of reverse and forward primers and 0.2 µl of Rox in each well. The primers used are listed in Supplementary Table S2.

### Statistical analysis

Data were analysed with R software (version 4.2.3). mRNA expression levels of the genes and LASSO-Cox coefficients were used to construct the HGSscore. The hazard ratio (HR) in the forest plot was determined via univariate Cox and multivariate Cox regression analyses. K‒M curves and log-rank tests were used for survival analysis. Differences between groups were assessed using the Wilcoxon test. The level of statistical significance was 0.05.

## Results

### Prognostic value of lipid homeostasis and identification of HGSscore

**Figure 1:**
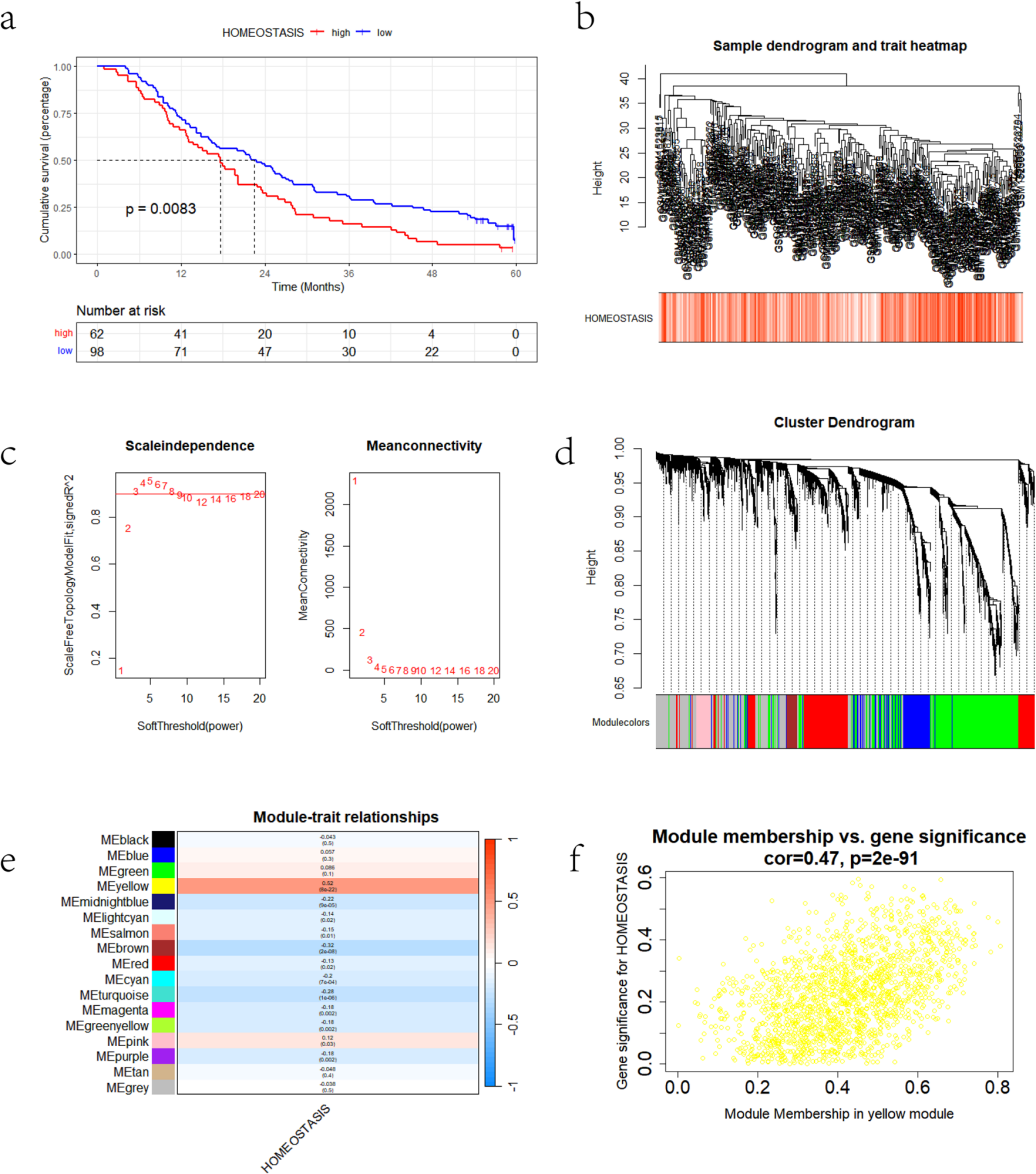
WGCNA algorithm for the GSE62254 cohort. (**a**) Kaplan–Meier OS curve showing that lipid homeostasis was significantly associated with worse GC prognosis. (**b**) Clustering dendrogram of samples from WGCNA. (**c**) The optimal soft threshold for the GSE62254 cohort. (**d**) Clustering dendrogram of all genes. (**e**) Heatmap of the correlations between the modules and lipid homeostasis. (**f**) Correlations between genes within the yellow module and lipid homeostasis.

**Figure 2:**
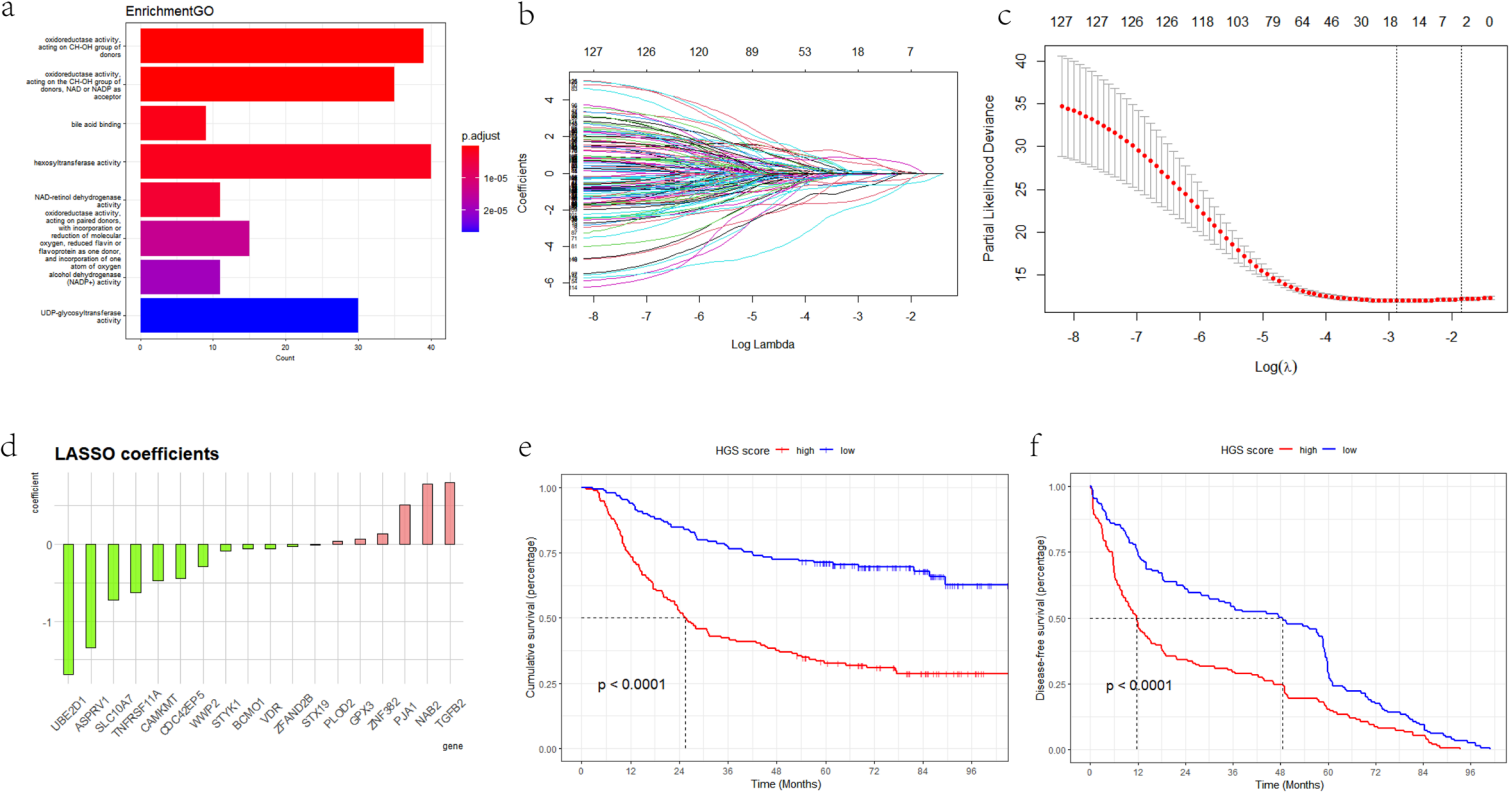
Establishment of HGSscore in the GSE62254 cohort. (**a**) Gene Ontology (GO) enrichment for genes in the yellow module. (**b**) LASSO coefficient profiles of lipid homeostasis-related prognostic genes. (**c**) Tenfold cross-validation for penalty parameter λ selection in the LASSO model. (**d**) LASSO coefficients of 18 selected genes. (**e**) K‒M OS curve for patients in the HGSscore-high and -low groups. (**f**) Kaplan–Meier DFS curve for patients in the HGSscore-high and -low groups.

The expression pattern of genes related to lipid homeostasis was assessed based on the transcriptome profile from GSE62254 using ssGSEA. The association between lipid homeostasis and 5-year OS in patients with GC was evaluated by using Kaplan‒Meier curves. We found that lipid homeostasis was significantly associated with 5-year OS (Fig. 1a). WGCNA was applied to construct a gene coexpression network to select the lipid homeostasis-related module from the whole transcriptome profile (Fig. 1b). The optimal soft threshold was set to three to achieve a scale-free network (Fig. 1c). Seventeen modules were identified. The yellow module correlated with lipid homeostasis the most (Fig. 1d–f). The genes within the yellow module were enriched in oxidoreductase activity, hexosyltransferase activity, and UDP-glycosyltransferase activity (Fig. 2a). Univariate Cox (Supplementary Table S3) and LASSO-Cox (Fig. 2b,c) analyses were used to identify highly prognosis-related genes. Finally, 18 genes and their coefficients were extracted for further study. We found that *TGFB2, NAB2, PJA1, ZNF382, GPX3*, and *PLOD2* were risk factors for GC; the other factors were protective factors for GC (Fig. 2d).

HGSscore for each patient was calculated using the mRNA expression level of the genes and their LASSO coefficients. Then, the GSE62254 dataset was divided into HGSscore-high and -low groups using the median HGSscore as the cut-off. K‒M OS and DFS curves demonstrated that the two groups had significantly different prognoses (Fig. 2e,f).

### HGSscore and clinical characteristics in the training cohort

**Figure 3:**
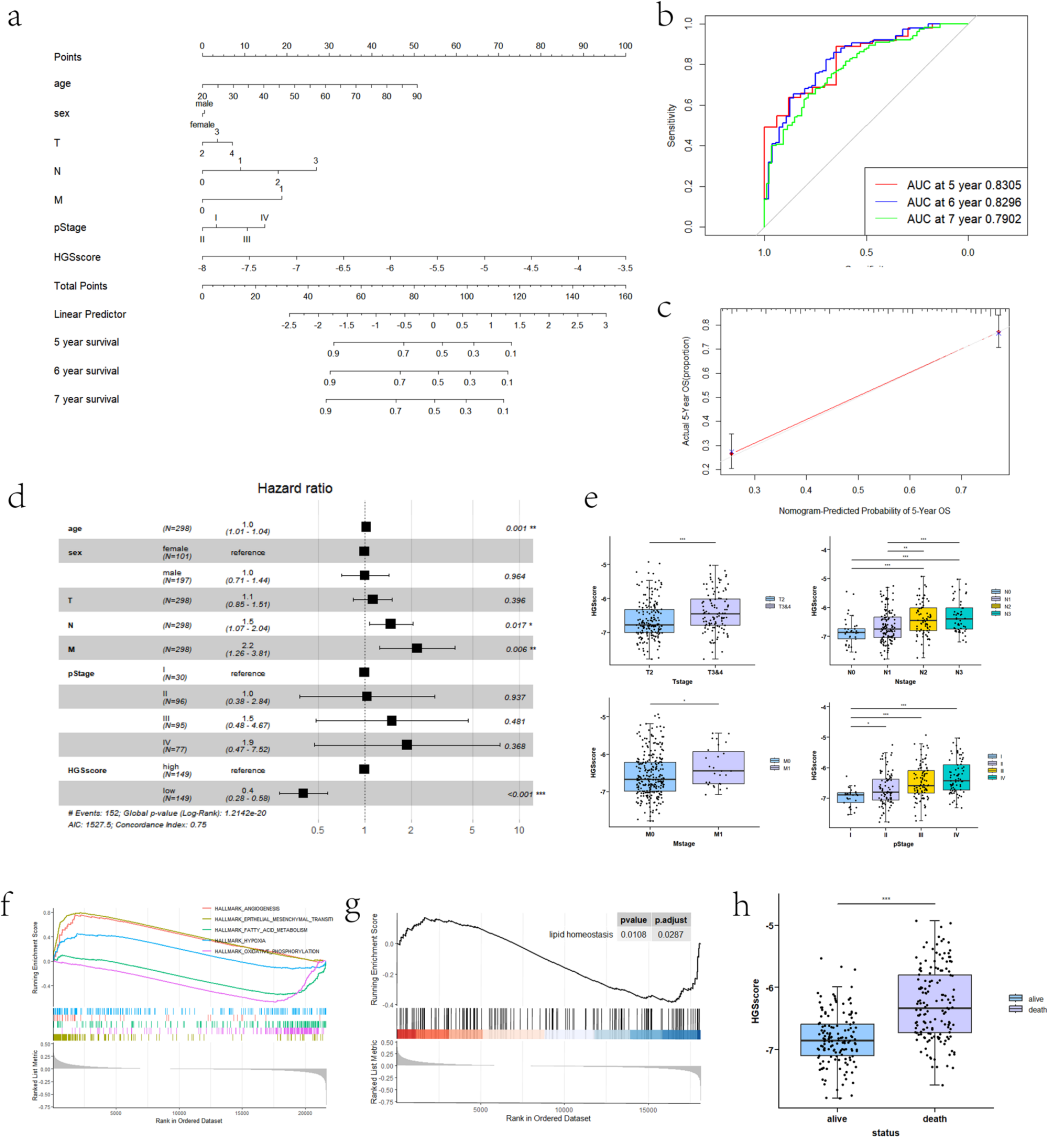
Validation of HGSscore in the GSE62254 cohort. (**a**) Nomogram based on HGSscore and clinical characteristics. (**b**) ROC curves at 5, 6, and 7 years. (**c**) Calibration curve for five-year OS. (**d**) Forest plot for multiple Cox regression coefficients, including HGSscore and clinical characteristics. (**e**) Correlations between HGSscore and clinical characteristics. (**f**,**g**) GSEA of several cancer hallmarks and lipid homeostasis pathways based on HGSscore-DEGs. (**h**) HGSscore of surviving and deceased groups.

A nomogram was developed based on clinical characteristics and HGSscore to predict the 5-, 6-, and 7-year OS of patients with GC (Fig. 3a). The areas under the ROC curves were 0.8305, 0.8296, and 0.7902, respectively (Fig. 3b). Calibration curves revealed great consistency between the nomogram-predicted OS and actual OS (Fig. 3c, Supplementary Fig. S1). Cox multivariate regression demonstrated that HGSscore was an independent risk factor for GC prognosis (Fig. 3d). Subsequently, correlations between HGSscore and pathological characteristics revealed that HGSscore was significantly associated with advanced TNM and clinical stage (Fig. 3e). GSEA of the HGSscore-high cohort revealed that the DEGs are related to cancer hallmark pathways, including angiogenesis, EMT, FA (fatty acid) metabolism, HYPOXIA, and OXPHOS, as well as the lipid homeostasis pathway (Fig. 3f,g). Finally, we found that patients with higher HGSscore often had worse prognoses (Fig. 3h).

### Validation of HGSscore in the TCGA and GEO cohorts

**Figure 4:**
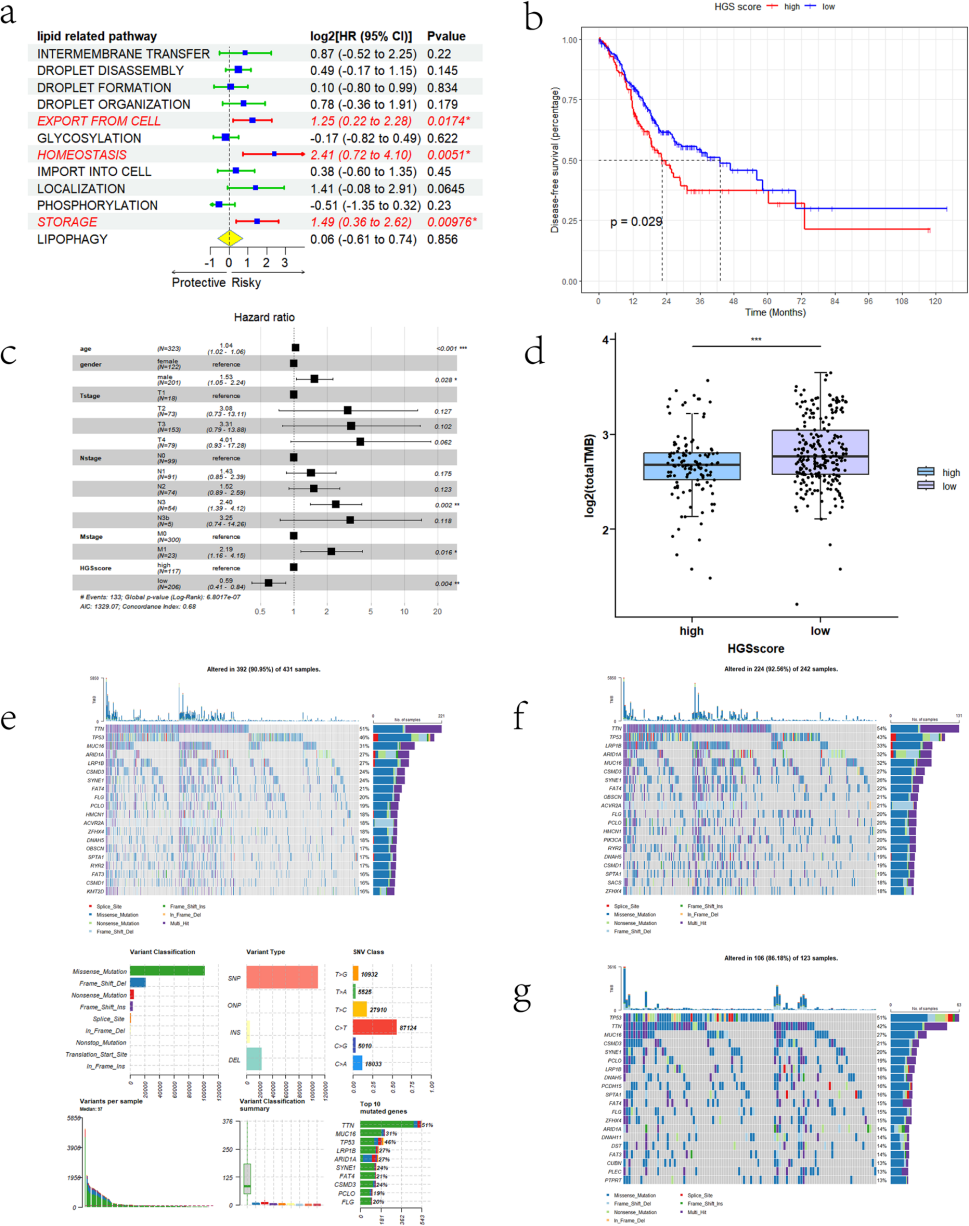
Validation of lipid homeostasis and HGSscore in the TCGA-STAD cohort. (**a**) Forest plot for univariate Cox regression of 12 lipid metabolism pathways. (**b**) K‒M OS curve for patients in the HGSscore-high and -low groups. (**c**) Forest plot for multivariate Cox regression, including HGSscore and clinical characteristics. (**d**) Comparison of total TMB in the HGSscore-high and -low groups. (**e**) Global landscapes of somatic mutations in TCGA-STAD cohort. (**f**,**g**) Top 20 somatic mutations in the HGSscore-high and -low groups.

To validate the prognostic value of HGSscore, we evaluated two independent cohorts (TCGA-STAD and GSE84437). In the TCGA-STAD cohort, lipid homeostasis was the riskiest pathway (HR = 2.41, p = 0.0051) among the 12 lipid-related pathways (Fig. 4a). K‒M curves demonstrated a significant difference in OS between patients in the HGSscore-high and -low groups (Fig. 4b). Cox multivariate regression also revealed that HGSscore was an independent risk factor for patient survival in patients with GC (Fig. 4c). Notably, we found that patients with higher HGSscore had lower TMBs (Fig. 4d), which indicated worse ICI response in this population. To extend the relationship between HGSscore and TMB, a comprehensive landscape of TMB in TCGA-STAD was constructed. We found that missense mutations dominated the variant subtypes in GC and that C>T single nucleotide polymorphisms accounted for more than 50% of the variant subtypes (Fig. 4e). Among the top 20 mutated genes, *TTN, TP53, MUC16, ARID1A*, and *LRP1B* were the most frequently mutated (Fig. 4e). We further analysed differences in TMB between the HGSscore-high and HGSscore-low groups and found that *TP53* was frequently mutated in the HGSscore-high subgroup (Fig. 4f) and that the TMB profile in the HGSscore-low subgroup was similar to that in the general population (Fig. 4g).

HGSscore also showed great prognostic value in the GSE84437 cohort (Supplementary Fig. S2). K‒M curves revealed that patients in different groups had completely different prognoses (p < 0.001).

### Mechanism of HGSscore in the tumour microenvironment

**Figure 5:**
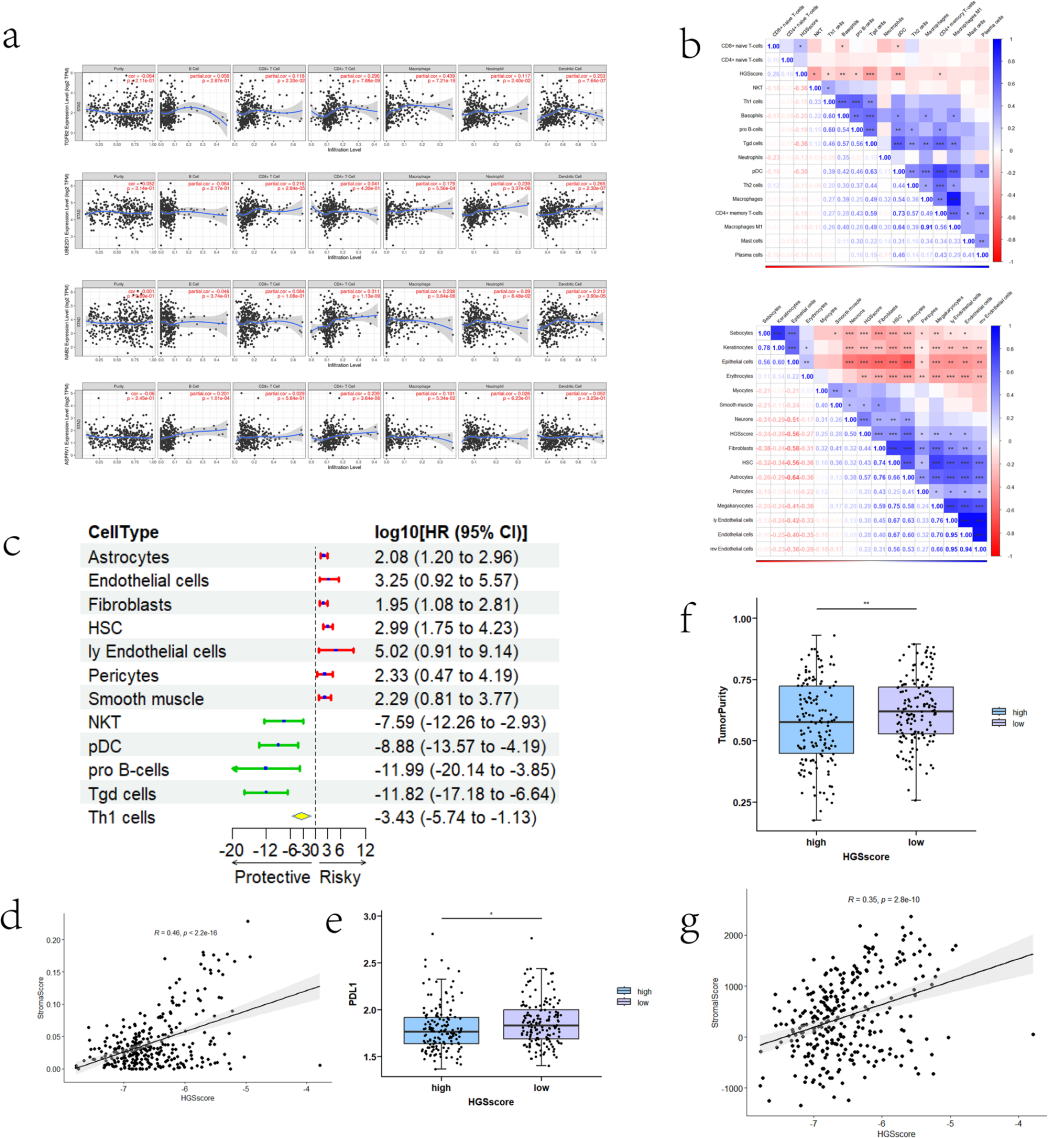
Correlations between HGSscore and tumour microenvironment components. (**a**) Immune cell infiltration predicted by TIMER. (**b**) Heatmaps displaying the correlation between HGSscore and various immune or stromal cells according to the xCell dataset. (**c**) Hazard ratio of 12 selected immune and stromal cells. (**d**) The correlation between the stromal score (xCell) and HGSscore. (**e**) Comparison of PDL1 expression in the HGSscore-high and -low groups. (**f**) Association between HGSscore and tumour purity according to the ESTIMATE database. (**g**) The correlation between the stromal score (ESTIMATE) and HGSscore.

Three immune/stromal cell infiltration predictive tools were used to reveal the potential mechanism of HGSscore within the TME. Specifically, TIMER was used to reveal the correlation between a single gene in HGSscore and immune cell infiltration. The highest risk and protective genes (*TGFB2, NAB2, UBE2D1,* and *ASPRV1*) in HGSscore were also analysed. *TGFB2* is related to macrophage infiltration and that *NAB2* is associated with CD4+ T-cell infiltration. *UBE2D1* correlates significantly with DC infiltration, and ASPRV1 is related to CD4+ T-cell infiltration (Fig. 5a).

Additionally, the xCell algorithm was applied to outline relationships between various immune/stromal cells and HGSscore (Fig. 5b). For immune cell infiltration, HGSscore was found to be negatively associated with NKT, Th1 cells, Tgd cells, basophils, pro-B cells, CD4+ memory T cells, and pDCs and positively related to CD8+ naive T cells in the TME. The correlations between HGSscore and stromal cells were much more complex than those between HGSscore and stromal cells, and HGSscore correlated with many stromal cells, such as epithelial cells and fibroblasts. Moreover, univariate Cox regression was used to evaluate the prognostic value of twelve immune/stromal cells (Fig. 5c). Among these selected cells, ly endothelial cells (lymphatic endothelial cells) were the highest risk factor (HR = 5.02) for GC, whereas pro-B cells were the most protective factor (HR = − 11.99). Spearman correlation analysis revealed that HGSscore correlated positively with the stromal score according to the xCell dataset (Fig. 5d). By comparing *PDL1* mRNA levels, we found that *PDL1* was lower in the HGSscore-high group (Fig. 5e).

Finally, ESTIMATE was used to assess tumour purity in the TME. The Wilcoxon test showed that tumour purity was slightly greater in the HGSscore-low subgroup than in the HGSscore-high subgroup (Fig. 5f). Interestingly, correlation analysis indicated that HGSscore was positively related to the stromal score determined via the ESTIMATE tool (Fig. 5g).

### Relationship between HGSscore and chemotherapeutic efficacy

**Figure 6:**
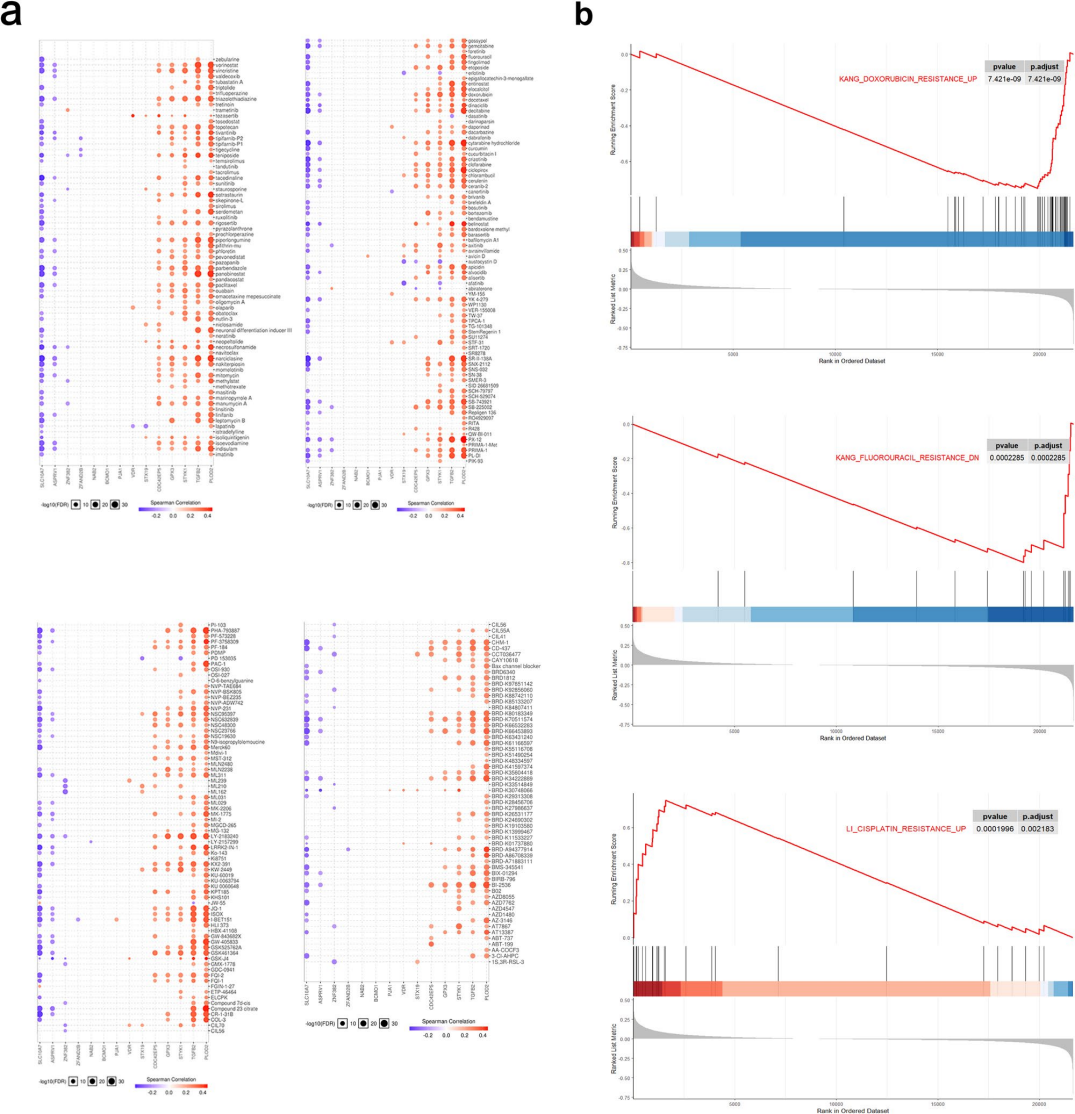
The correlation between HGSscore and chemotherapeutic efficacy. (**a**) Spearman correlation between lipid homeostasis-related genes and chemotherapeutic efficacy. (**b**) GSEA of doxorubicin, fluorouracil and cisplatin resistance based on individual gene sets.

The Cancer Therapeutics Response Portal (CTRP) and Genomics of Drug Sensitivity in Cancer (GDSC) databases were used to demonstrate the correlation between HGSscore and chemotherapeutic efficacy. We found that eight genes, *SLC10A7, ASPRV1, ZNF382, CDC42EP5, GPX3, STYK1, TGFB2*, and *PLOD2,* were significantly associated with the chemotherapeutic effect of GC on CTRP. Among these genes, *SLC10A7, ASPRV1*, and *ZNF382* are related to chemotherapy response; *CDC42EP5*, *GPX3, STYK1, TGFB2*, and *PLOD2* are related to chemotherapy resistance (Fig. 6a). In GDSCs, *PLOD2* is considered to be linked to drug resistance, and *SLC10A7* and *ASPRV1* are linked to drug response (Supplementary Fig. S3). GSEA of gene sets related to doxorubicin, cisplatin, and fluorouracil resistance revealed that patients in the HGSscore-high cohort had a satisfactory response to doxorubicin, though they were resistant to cisplatin and fluorouracil therapy (Fig. 6b).

### Disruption of the lipid homeostasis pathway mediated by exogenous lipids

To determine the effect of lipid homeostasis on GC, we detected relative cell viability and expression of several mRNAs in the SGC7901 cell line treated with fatty acids. According to the results of the cell viability assay, SGC7901 cells exposed to lipids exhibited reduced cell viability compared to those in the ethanol group (Supplementary Fig. S4a). RT‒qPCR revealed that *ASPRV1* was upregulated after treatment; the other five genes were downregulated (Supplementary Fig. S4b). Taken together, these findings suggest that lipid homeostasis has an impact on GC cells.

## Discussion

In the last decade, treatment of gastric cancer has progressed continuously, but prognostic information has not been updated much. Lipid homeostasis is widely reported to be critical for GC growth^12^, metastasis^13^, chemotherapeutic resistance^14^, and immunity^15^. However, studies that highlight the correlation between lipid homeostasis and GC prognosis from both clinical and molecular biological perspectives are limited.

In the present study, we found lipid homeostasis to be a risk factor for GC prognosis, and a GC prognostic model was constructed based on eighteen lipid homeostasis-related genes. As expected, this model demonstrated satisfactory value in risk stratification, survival prediction, tumour immunity, and chemotherapeutic response. Recently, several studies have explored the clinical value of altered lipidomic profiles in tumour tissue or serum^16–18^ and revealed that abnormal lipid metabolites may lead to poor clinical outcomes. In colorectal cancer^16^, the triacylglycerol (TG) profile is significantly associated with disease-free survival and lymphovascular invasion. In addition, lipogenic genes such as fatty acid synthase (FASN) were found to be significantly upregulated in CRC patients and risk factors for patient survival. In summary, lipids not only play a prominent role in clinical progression of cancer but also predict the prognosis of patients precisely.

The TME consists of various cell types embedded in the extracellular matrix, including cancer cells, immune cells, and stromal cells, which contribute to an immunosuppressive environment during the progression of cancer. Recent studies have elucidated the important role of lipids in remodelling the TME. In this study, we found that the HGSscore was negatively associated with infiltration of immune cells, including CD8+ T cells, DCs, NKT cells, and Th1 cells. Accumulated evidence has demonstrated that lipids can promote CD8+ T-cell exhaustion and further undermine CD8+ T-cell function^19,20^. In 2020, Alison E. Ringel reported that high-fat diet-induced obesity could impair CD8+ T-cell function and promote tumour growth^21^, which proved that systemic disruption of lipid homeostasis could affect intratumoral immunity. In addition, fatty acids in tumour-derived exosomes induce immune dysfunction in DCs via PPAR-alpha activation and facilitate immune evasion in colon cancer^22^. Another study reported that tumours impair DC function through induction of the ER stress response and disruption of lipid metabolic homeostasis^23^. Wenshu Tang revealed that NKT cell function is inhibited by mTORC1/SREBP2-induced cholesterol accumulation and subsequently promotes progression of obesity-related HCC^24^. In inflammatory disease, investigators have shown that cholesterol homeostasis is essential for Th1 cells to accomplish effector-regulatory switching, and perturbations in cholesterol flux are risk factors for RA^25^.

Moreover, this study showed that lipid homeostasis is strongly associated with stromal cell infiltration. Fibroblasts in the TME are also known as carcinoma-associated fibroblasts (CAFs) and are critically associated with tumorigenesis and tumour progression by boosting angiogenesis, cancer cell proliferation, and invasion. Gui-Qi Zhu reported that CD36+ CAFs in hepatocellular carcinoma exhibit high lipid metabolism and secrete macrophage migration inhibitory factor via the lipid peroxidation/p38/CEBPs axis, leading to an immunosuppressive TME and tumour stemness^26^. Additionally, after reprogramming of lipid metabolism, lipid metabolites secreted by CAFs were proven to promote CRC migration in a CD36-dependent manner^27^. Interestingly, prostaglandin E2 (PGE2), a common lipid inflammatory factor, is considered to be a key molecule in CAFs that regulates breast cancer growth and metastasis^28^. In our study, lipid homeostasis was shown to facilitate CAF infiltration, which is a risk factor for GC prognosis.

Endothelial cells are the basis of blood and lymphatic vessels in the TME. However, these vessels also exhibit abnormalities in their structure and function, which results in a hostile tumour microenvironment and tumour metastasis^29^. Vincent Geldhof identified a distinct endothelial cell type, LIPEC, in breast cancer, which was characterized by an active lipid metabolism pathway^30^. Patients with more LIPEC may be considered to benefit from metformin treatment. LPA and S1P are two critical lipid mediators for tumour angiogenesis. A recent review concluded that LPA and S1P are crucial for endothelial cell migration and promoting tumour invasion and metastasis via specific GPCR pathways^31^.

Furthermore, we found that lipid homeostasis correlated with the tumour mutation burden (TMB) and chemotherapeutic efficacy. An analysis involving 206 GC patients and 435 cancer-related genes was conducted to explore the prognostic value of the TMB and mutational signatures in GC^32^. The data from this research showed that the TMB-high subgroup had a relatively worse prognosis, greater lipid metabolism activity and more frequent metabolic disorders than the TMB-low subgroup, indicating that immunotherapy in combination with metabolic inhibitors may be a novel treatment for GC. Nevertheless, our study revealed that *TP53* was the most frequently mutated gene in the TMB-high cohort, which demonstrated a notable potential for GC management.

Accumulating evidence has indicated that lipids participate in drug resistance through multiple mechanisms. Recently, investigators have shown that free fatty acids generated by cancer-associated adipocytes (CAAs) can induce antiangiogenic drug (AAD) resistance^33,34^, and AADs, such as bevacizumab, are widely used in various cancers. Specifically, hypoxia-related lipid metabolic reprogramming in adipocytes was found to be triggered by hypoxia, which revealed the intensive communication between these two cancer hallmarks. Carnitine palmitoyltransferase (CPT), the key enzyme for fatty acid transportation, not only promotes AAD resistance but also facilitates oxaliplatin resistance; oxaliplatin is a fundamental chemical in most chemotherapy regimens^14,33^. In addition, ferroptosis is a novel cell death pathway characterized by accumulation of lipid ROS^35^. Several studies have demonstrated that ferroptosis can regulate acquired chemotherapy resistance in gastrointestinal cancers^36,37^ through lipid-related enzymes and proteins that play pivotal roles in this process. In this study, we found that lipid homeostasis correlated significantly with multidrug resistance in GC. In particular, *PLOD2* is reported to stimulate the cancer stem cell phenotype and cisplatin resistance in cancer^38^. TGFB2 is a common cytokine involved in intercellular communication and FOLFOX resistance^39^, and we found that it was strongly associated with chemoresistance.

## Conclusion

In this study, we constructed a novel gene signature based on 18 lipid homeostasis-related genes that performed well in predicting the prognosis of GC. Subsequently, the correlation between lipid homeostasis and immune/stromal cell infiltration was illustrated, which might be the potential mechanism through which lipids act in the TME. Finally, we identified several genes of HGSscore that were associated with chemotherapeutic response or resistance. Although further prospective studies in larger cohorts are needed to validate the utility of HGSscore, its ability to predict patient prognosis and guide individualized chemotherapy should not be ignored.

### Supplementary Information

Supplementary Figure S1.

Supplementary Figure S2.

Supplementary Figure S3.

Supplementary Figure S4.

Supplementary Table S1.

Supplementary Table S2.

Supplementary Table S3.

## Data Availability

All data are publicly available in this study. GSE84437 and GSE62254 can be accessed at https://www.ncbi.nlm.nih.gov/gds. TCGA-STAD can be accessed at https://portal.gdc.cancer.gov/projects/TCGA-STAD. MsigDB can be accessed at https://www.gsea-msigdb.org/gsea/msigdb. And TIMER can be accessed at https://cistrome.shinyapps.io/timer/.
