# Supplementary material for: Identification of a lipid homeostasis-related gene signature for predicting prognosis, immunity, and chemotherapeutic effect in patients with gastric cancer
Source: Sci Rep. 2024 Feb 5;14:2895. doi: 10.1038/s41598-024-52647-7 (PMC10844315; doi:10.1038/s41598-024-52647-7)
Supplement: Supplementary file 2 — Supplementary Figure S2. [file 41598_2024_52647_MOESM2_ESM.pdf]

Identification of a Lipid Homeostasis Related Genes Signature in Predicting the Prognosis, Immunity, and Chemotherapeutic Effect for Patients with Gastric Cancer

Chao Li[1], Zhen Xiong[1], Jinxin Han[1], Weiqi Nian[2], Zheng Wang[1], Kailin Cai[1], Jinbo Gao[1], Guobin Wang[1], Kaixiong Tao[1], Ming Cai[1*]

Supplementary figure S2

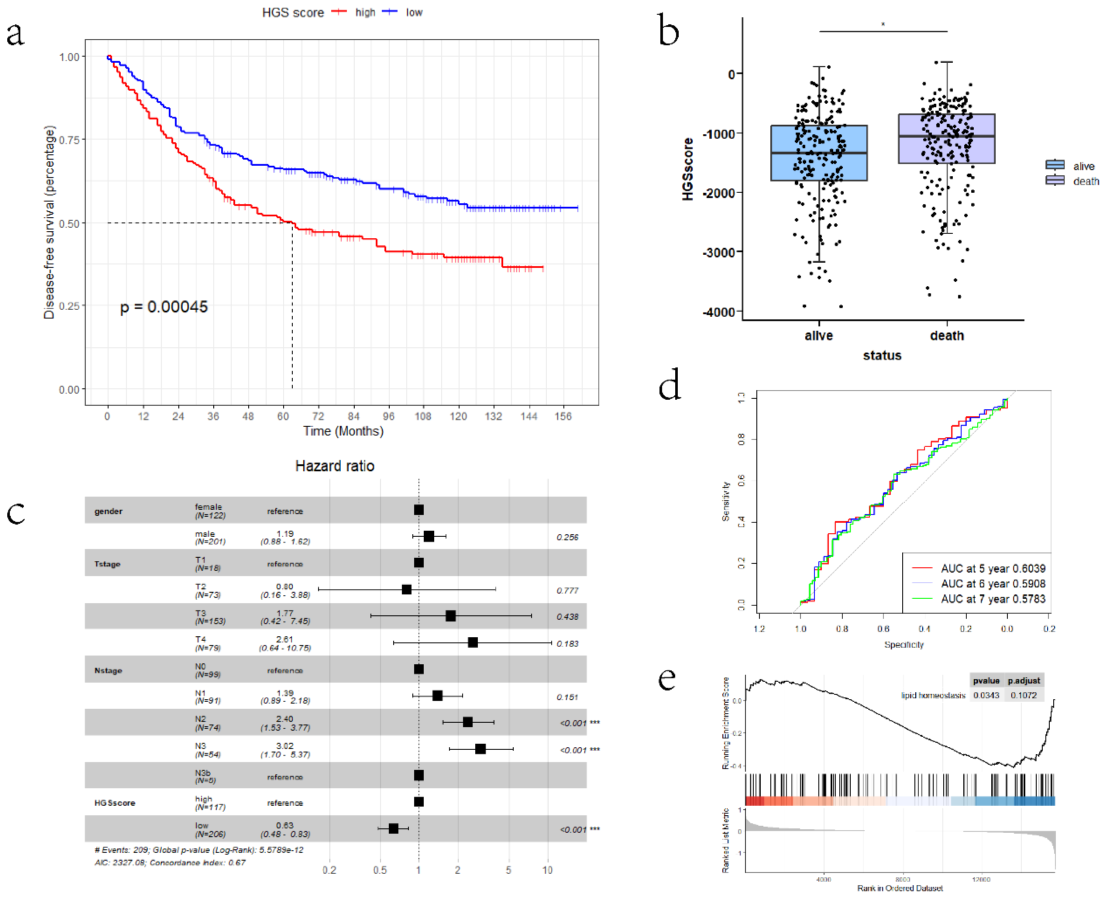

Validation of HGSscore in the GSE84437 cohort. (a) Kaplan–Meier OS curve for patients in the HGSscore-high and -low groups. (b) The HGSscore was greater in patients with worse prognosis. (c) A forest plot for multivariate Cox regression showed that HGSscore was an independent risk factor. (d) ROC curves at 5, 6 and 7 years. (e) GSEA revealed that lipid homeostasis was significantly enriched.
