# Supplementary material for: Identification of a lipid homeostasis-related gene signature for predicting prognosis, immunity, and chemotherapeutic effect in patients with gastric cancer
Source: Sci Rep. 2024 Feb 5;14:2895. doi: 10.1038/s41598-024-52647-7 (PMC10844315; doi:10.1038/s41598-024-52647-7)
Supplement: Supplementary file 3 — Supplementary Figure S3. [file 41598_2024_52647_MOESM3_ESM.pdf]

Identification of a Lipid Homeostasis Related Genes Signature in Predicting the Prognosis, Immunity, and Chemotherapeutic Effect for Patients with Gastric Cancer

Chao Li[1], Zhen Xiong[1], Jinxin Han[1], Weiqi Nian[2], Zheng Wang[1], Kailin Cai[1], Jinbo Gao[1], Guobin Wang[1], Kaixiong Tao[1], Ming Cai[1]*

Supplementary figure S3

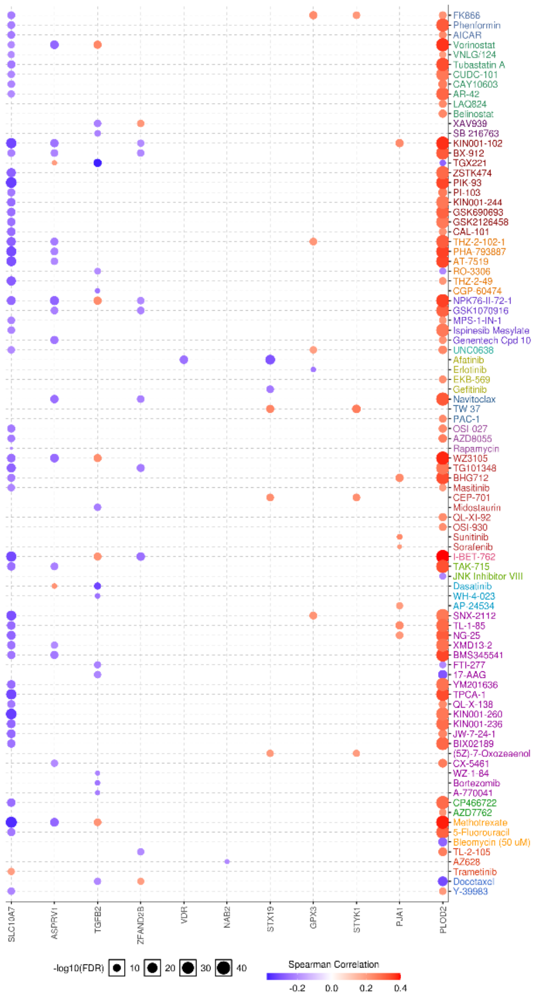

Chemotherapeutic response predicted by the GDSC platform.
